# Supplementary material for: HIVprotI: an integrated web based platform for prediction and design of HIV proteins inhibitors
Source: J Cheminform. 2018 Mar 9;10:12. doi: 10.1186/s13321-018-0266-y (PMC5845081; doi:10.1186/s13321-018-0266-y)
Supplement: Supplementary file 2 — Additional file 2. Source code of HIVProtI web server. [file 13321_2018_266_MOESM2_ESM.zip › HIVprotI_Source-code/hivprotI/weka-3-6-6/doc/weka/core/matrix/EigenvalueDecomposition.html]

EigenvalueDecomposition


---


|  |  |  |
| --- | --- | --- |
| **Overview** \| **Package** \| **Class** \| **Tree** \| **Deprecated** \| **Index** \| **Help** \| **Weka's home** | |  |
| **PREV CLASS**   **NEXT CLASS** | **FRAMES**    **NO FRAMES**     **All Classes** |  |
| SUMMARY: NESTED \| FIELD \| CONSTR \| METHOD | DETAIL: FIELD \| CONSTR \| METHOD |  |


---


## weka.core.matrix Class EigenvalueDecomposition

```
java.lang.Object
  weka.core.matrix.EigenvalueDecomposition
```

**All Implemented Interfaces:**: java.io.Serializable, RevisionHandler

---

``` public class EigenvalueDecomposition extends java.lang.Object implements java.io.Serializable, RevisionHandler ```

Eigenvalues and eigenvectors of a real matrix.

If A is symmetric, then A = V\*D\*V' where the eigenvalue matrix D is diagonal
and the eigenvector matrix V is orthogonal. I.e. A =
V.times(D.times(V.transpose())) and V.times(V.transpose()) equals the
identity matrix.

If A is not symmetric, then the eigenvalue matrix D is block diagonal with
the real eigenvalues in 1-by-1 blocks and any complex eigenvalues, lambda +
i\*mu, in 2-by-2 blocks, [lambda, mu; -mu, lambda]. The columns of V
represent the eigenvectors in the sense that A\*V = V\*D, i.e. A.times(V)
equals V.times(D). The matrix V may be badly conditioned, or even singular,
so the validity of the equation A = V\*D\*inverse(V) depends upon V.cond().

Adapted from the JAMA package.

**Version:**
:   $Revision: 1.4 $

**Author:**
:   The Mathworks and NIST, Fracpete (fracpete at waikato dot ac dot nz)

**See Also:**: Serialized Form

---

**Constructor Summary**

`EigenvalueDecomposition(Matrix Arg)`
  
          Check for symmetry, then construct the eigenvalue decomposition


| **Method Summary** | |
| --- | --- |
| `Matrix` | `getD()`             Return the block diagonal eigenvalue matrix |
| `double[]` | `getImagEigenvalues()`             Return the imaginary parts of the eigenvalues |
| `double[]` | `getRealEigenvalues()`             Return the real parts of the eigenvalues |
| `java.lang.String` | `getRevision()`             Returns the revision string. |
| `Matrix` | `getV()`             Return the eigenvector matrix |

**Methods inherited from class java.lang.Object**

`equals, getClass, hashCode, notify, notifyAll, toString, wait, wait, wait`

**Constructor Detail**

### EigenvalueDecomposition

```
public EigenvalueDecomposition(Matrix Arg)
```

:   Check for symmetry, then construct the eigenvalue decomposition

    **Parameters:**: `Arg` - Square matrix


**Method Detail**

### getV

```
public Matrix getV()
```

:   Return the eigenvector matrix

    :   **Returns:**: V

---


### getRealEigenvalues

```
public double[] getRealEigenvalues()
```

:   Return the real parts of the eigenvalues

    :   **Returns:**: real(diag(D))

---


### getImagEigenvalues

```
public double[] getImagEigenvalues()
```

:   Return the imaginary parts of the eigenvalues

    :   **Returns:**: imag(diag(D))

---


### getD

```
public Matrix getD()
```

:   Return the block diagonal eigenvalue matrix

    :   **Returns:**: D

---


### getRevision

```
public java.lang.String getRevision()
```

:   Returns the revision string.

    :   **Specified by:**: `getRevision` in interface `RevisionHandler`
    :   **Returns:**: the revision


---


|  |  |  |
| --- | --- | --- |
| **Overview** \| **Package** \| **Class** \| **Tree** \| **Deprecated** \| **Index** \| **Help** \| **Weka's home** | |  |
| **PREV CLASS**   **NEXT CLASS** | **FRAMES**    **NO FRAMES**     **All Classes** |  |
| SUMMARY: NESTED \| FIELD \| CONSTR \| METHOD | DETAIL: FIELD \| CONSTR \| METHOD |  |


---
